# Non-Photochemical Quenching in Cryptophyte Alga *Rhodomonas salina* Is Located in Chlorophyll a/c Antennae

**DOI:** 10.1371/journal.pone.0029700

**Published:** 2012-01-03

**Authors:** Radek Kaňa, Eva Kotabová, Roman Sobotka, Ondřej Prášil

**Affiliations:** Institute of Microbiology, Czech Academy of Sciences, Třeboň, Czech Republic; University of Hyderabad, India

## Abstract

Photosynthesis uses light as a source of energy but its excess can result in production of harmful oxygen radicals. To avoid any resulting damage, phototrophic organisms can employ a process known as non-photochemical quenching (NPQ), where excess light energy is safely dissipated as heat. The mechanism(s) of NPQ vary among different phototrophs. Here, we describe a new type of NPQ in the organism *Rhodomonas salina*, an alga belonging to the cryptophytes, part of the chromalveolate supergroup. Cryptophytes are exceptional among photosynthetic chromalveolates as they use both chlorophyll a/c proteins and phycobiliproteins for light harvesting. All our data demonstrates that NPQ in cryptophytes differs significantly from other chromalveolates – e.g. diatoms and it is also unique in comparison to NPQ in green algae and in higher plants: (1) there is no light induced xanthophyll cycle; (2) NPQ resembles the fast and flexible energetic quenching (qE) of higher plants, including its fast recovery; (3) a direct antennae protonation is involved in NPQ, similar to that found in higher plants. Further, fluorescence spectroscopy and biochemical characterization of isolated photosynthetic complexes suggest that NPQ in *R. salina* occurs in the chlorophyll a/c antennae but not in phycobiliproteins. All these results demonstrate that NPQ in cryptophytes represents a novel class of effective and flexible non-photochemical quenching.

## Introduction

Photosynthetic organisms are often exposed to varying environmental conditions, such as excessive irradiation. Over-excitation by surfeit light can produce harmful reactive oxygen intermediates detrimental to pigments, proteins and lipids [1], [2]. Several protective mechanisms can be stimulated when light absorption exceeds its utilisation in photosynthesis. One of these, non-photochemical quenching (NPQ), is a feed-back regulatory mechanism in which excessive light irradiation is dissipated as heat (reviewed in [3], [4]). As the process of NPQ involves the de-excitation of chlorophyll molecules from their exited states, NPQ is usually detected indirectly by analysing chlorophyll fluorescence [5], rather than directly by monitoring heat emissions [6].

Mechanism of NPQ is best characterized in higher plants [3], [7]. The process of energy dissipation in NPQ is triggered by low pH in the thylakoid lumen and modulated by several factors including zeaxanthin [8] and the PsbS protein [9], [10]. The search for the molecular photophysical mechanism of NPQ remains unresolved as several quenching mechanism have been already suggested including quenching by lutein [11], chlorophyll to zeaxanthin charge transfer quenching [12], by chlorophyll pairs [13] or quenching by excitonic carotenoid–chlorophyll states [14]. The understanding of NPQ is complex as several processes, each with different kinetics, are involved. On the beginning, the fast energetic quenching (qE) is triggered by lumen acidification, that is further stimulated under prolonged irradiation by zeaxanthin [15]. Long-term exposition (hours and days) to excessive irradiation then finally results in photoinhibitory quenching - qI which exact mechanism is still matter of debate [4], [16].

Although mainly documented in plants, NPQ has also been studied in several oxygenic phototrophs; mosses, algae and cyanobacteria. The basic principle of NPQ, the safe dissipation of excessive light irradiation as heat, is identical across all organisms; however, crucial differences exist in its regulation and structural mechanisms. For example, NPQ in diatoms [17] is located in fucoxanthin–chlorophyll *a/c* antennae [18], [19], that are non-homologous to chlorophyll *a/b* antennae of higher plants. In addition to the structure differences, these two antennae also differ in their sensitivity to protonation, as only chlorophyll *a/b* antennae are able to be reversibly protonated [20], [21] but such a effect is questionable in diatoms [17], [22]. On the regulatory level, PsbS is known to be active in NPQ of higher plants, but not in diatoms [23], [24] or green algae [25]. Instead another group of light-harvesting proteins from the LHCSR (formerly LI818) family, which are missing in higher plants [25] are involved in NPQ in green algae and in diatoms [26], [27]. Additional differences exist also in the ability of transthylakoid ΔpH to trigger NPQ; the capacity of ΔpH to induce quenching is decreased in some green algae when compared to higher plants [28]. There are also different xanthophyll cycles, a violaxanthin cycle found in green algae [29] and a diadinoxanthin cycle in diatoms [30]. Furthermore, in cyanobacteria completely different mechanism of NPQ, regulated by the OCP protein, operates in the phycobilisomes [31].

Compared to higher plants, the understanding of NPQ in various algal groups is still much more fragmented or missing completely. This is especially true for chromalveolate algae involving diatoms, brown algae and cryptophytes [32]. The chromalveolate group is thought to have originated from a secondary endosymbiosis, when a chimeric organism was formed from two eukaryotic cells, a non-photosynthetic host and a photosynthetic endosymbiont of red algal origin [33]. Cryptophytes are exceptional among photosynthetic chromalveolates [32] as they are the only phototrophs to use for light harvesting both membrane-bound chlorophyll *a/c* proteins and phycobiliproteins that are firmly embedded in the thylakoid lumen [34]. Thus cryptophytes represent a unique evolutionary intermediate between ancestor of all chromalveolates - red algae, which contain phyobiliproteins but lack chlorophyll *c*, and diatoms, that have diversified more “recently” from their red algae ancestor and which contain chlorophyll *c* but not phycobiliproteins [23]. Moreover, the light harvesting antennae found in the cryptophytic thylakoid membrane are formed by unique chlorophyll *a/*c proteins known as CAC antennae [35]. These proteins are distinct from chlorophyll *a/b* binding antennae of green algae and higher plants and also from chlorophyll *c* antennae of chromalveolates; this includes the peridinin-chlorophyll proteins of dinoflagellates and the fucoxanthin-chlorophyll proteins of diatoms [36], [37].

For chromalveolate algae, diatoms are almost the only model organism used for intensive studding of NPQ mechanism [38]. It has been already found that NPQ in diatoms is a pH-dependent process closely associated with the diadinoxanthin cycle [17], localised at either the fucoxanthin-chlorophyll proteins [19] or the PSII reaction centre [39]. Diatoms aside, few publications exist on NPQ activity in chromalveolates, these are limited to studies on brown algae [40], the recently discovered apicomplexan *Chromera velia* [41] and the all chromalveolate ancestor, red algae [42], [43]. The mechanism of NPQ in red algae is still rather enigmatic, we only know that non-photochemical quenching of fluorescence in red algae is a pH-dependent process, precise NPQ locus is not known [42], [43]. A possible role of energy dissipation in PSII reaction centre [43] and physical phycobilisome decoupling [44] have been already discussed as possible energy dissipation pathways.

Even less we know about protective mechanisms in cryptophytes. It has been shown that none of the usual xanthophyll cycle pigments (e.g. zeaxanthin, diadinoxanthin, diatoxanthin) are present at detectable level during stimulation of NPQ in *Guillardia Theta* [45]. Here we describe NPQ mechanism in the cryptophytic algae representative, *Rhodomonas salina* in all its details that allowed us to compare it with the same process in diatoms and in higher plants. All our results have demonstrated that NPQ in cryptophytes represents a novel class of effective non-photochemical quenching. We have showed that the process of NPQ in cryptophytes is not accompanied by the cycling of xanthophyll pigments in line with previous results and moreover its kinetics resembles the rapid and reversible energetic quenching (qE) found in higher plants. The similarity of NPQ in cryptophytes with qE of plants was further confirmed with isolated antennae complexes, that showed involvement of their protonation in the cryptophytic NPQ process. Therefore, NPQ in cryptophytes is localised to the membrane-bound CAC protein that can be triggered to quenching mode by lumen acidification.

## Materials and Methods

### Cell growth and sample treatment

The cryptophyte alga *Rhodomonas salina* (strain CCAP 978/27) was grown in an artificial seawater medium with f/2 nutrient addition. The cell suspension was bubbled with air in the temperature controlled bath (t = 18°C) and illuminated by dimmable fluorescence tubes with intensity 30 µmol m^−2^ s^−1^ (day-night cycle 12/12 hours) [46]. For high light treatment the cell suspension was exposed for 75 minutes to white light emitting diode array with intensity 1000 µmol m^−2^ s^−1^.

### Measurements of variable fluorescence

Variable chlorophyll a fluorescence was measured by kinetic modulated fluorometer FL-3000 (Photon System Instrument, Brno, Czech Republic). Chlorophyll a fluorescence was detected in the spectral range 690–710 nm. Samples were dark adapted for 20 minutes before applying low intensity measuring light (2 µmol m^−2^ s^−1^, 622 nm) for the detection of intrinsic fluorescence of the dark adapted sample (F_0_). Maximal fluorescence for the dark (F_M_) and light adapted sample (F_M_′) has been measured during 200 ms multiple turnover actinic flashes. The light dependency curves of the photochemical efficiency (the “Genty parameter” calculated as φ_PSII_ = (F_M_′-F_t_)/F_M_′)) and of non-photochemical quenching (calculated as NPQ = (F_M_-F_M_′)/F_M_)) were measured with fresh sample for each intensity of the actinic light.

### Kinetic spectroscopy

The kinetic changes in the whole fluorescence spectrum were measured with millisecond time resolution using the spectrometer SM-9000 (Photon System Instrument, Brno, Czech Republic) with absolute wavelength accuracy of 0.8 nm; relative resolution reflecting FWHM Δλ = 3 nm [47]; the dark current of the instrument was automatically subtracted before measurements. Samples were dark adapted for 20 minutes before measurements, the spectrum of maximal fluorescence in the dark F_M_(λ) has been detected 150 ms after triggering the blue (466 nm, 1100 µmol m^−2^ s^−1^) or green (520 nm, 500 µmol m^−2^ s^−1^) saturating flashes of 200 ms duration. Later, the blue or green actinic (500 µmol m^−2^ s^−1^) lights were applied by the FL-100 fluorometer (Photon System Instrument, Brno, Czech Republic) to induce the non-photochemical quenching. The spectra of maximal fluorescence in the light F_M_′(λ) were detected after 2 minutes of actinic irradiation. The spectrally resolved NPQ(λ) was calculated using the Stern-Volmer formalisms as NPQ(λ) = [F_M_(λ)-F_M_′(λ)]/[F_M_(λ)] for every wavelength.

### Measurement of photosynthetic rates

Photosynthetic carbon fixation (P_g_) was determined by incorporation of radioactive H^14^CO_3_^−^ and analyzed as described in [48]. The samples were incubated with the ^14^C isotope in the laboratory-built photosynthetron for 40 min at 18°C. The total dissolved CO_2_ in the media was determined by alkalinity titrations. Photosynthetic rate was normalized to chlorophyll *a* content determined from absorbance of a methanol extract.

### Determination of photosynthetic pigments by HPLC

The aliquots of algal suspension were collected on GF/F filters (Whatman, England), soaked overnight at −20°C in 100% methanol and subsequently disrupted using mechanical tissue grinder. Samples were kept in cold and darkness to minimize pigment degradation. Filter and cell debris were removed by centrifugation (12000 g, 15 min) and the extract was injected into the Agilent 1200 HPLC system equipped with the DAD detector. Pigments were separated on the Luna 3 µ C8 column (100×4.60 mm; Phenomenex) at 35°C using a linear gradient from 0.028 M ammonium acetate/methanol (20/80) to 100% methanol and with a flow rate set to 0.8 mL/min. Eluted pigments were identified according to absorbance spectra and the respective retention times.

### pH dependent quenching in isolated antennae proteins

To isolate native protein complexes ∼0.5 L of *R. salina* cell suspension in exponential growth phase was harvested and resuspended in a thylakoid buffer containing 25 mM Mes/NaOH, pH 6.5, 10 mM CaCl_2_, 10 mM MgCl_2_ and 5% glycerol. Cells were broken using EmulsiFlex C-5 (Avestin Inc., Canada) using two cycles with maximal pressure 150 MPa. Membranes were pelleted by 40 000 g, 20 min, washed once in the thylakoid buffer and resuspended in the same buffer at concentration ∼0.5 µg chlorophyll/µL. Membrane proteins were solubilised with dodecyl-β-maltoside (dodecyl-β-maltoside/chlorophyll = 20, w/w). CAC oligomers and supercomplexes of CAC protein and photosystems were isolated by ultracentrifugation (20 h, 75 000 g) in 15%–25% gradient of sucrose in the thylakoid buffer containing 80 µM dodecyl-β-maltoside.

The chlorophyll a fluorescence quenching on isolated CAC proteins were analyzed by FL 100 (Photon System Instrument, Brno, Czech Republic) at 150 µmol m^−2^ s^−1^ with continual stirring. Purified proteins were diluted by 20 mM HEPES (pH 8.0, 5 mM MgCl_2_, 5 mM CaCl_2_, 0.1 M NaCl, 10% glycerol) to reach ∼8 µM concentration of dodecyl-β-maltoside in the sample. Then, pH was decreased to 5.5 by titration of 5% HCl to induce quenching, that was finally recovered by addition of N,N′-dicyclohexyl-carbodiimide (DCCD), final concentration 200 µM.

### Analysis of protein complexes by 2D electrophoresis

Solubilised membrane proteins were prepared from ∼100 mL of cells as described above just using the thylakoids with 25% of glycerol. Protein complexes were analysed by clear-native electrophoresis (CN-PAGE) according to [49]. 4–12% gradient polyacrylamide gel was run using Hoefer miniVE system at 4°C in a cathode buffer containing 0.25 mM Tricine, 7.5 mM Bis-Tris/HCl, pH 7.0, 0.05% sodium deoxycholate, 0.02% dodecyl-β-maltoside and with an anode buffer containing 0.25 mM Bis-Tris/HCl, pH 7.0. Resulting gel was scanned in true colors by Canon CanoScan 8800F scanner. For spectroscopic analysis of photosynthetic complexes separated by CN-PAGE ‘green’ bands were cut out from the gel and their absorbance detected at room temperature by UV 3000 spectrophotometer (Shimadzu, Kyoto, Japan) with spectral bandwidth 1 nm. Fluorescence emission spectra were detected at room temperature by Aminco Bowman Series 2 spectrofluorometer (Thermo Fisher Scientific, USA) with 1 nm spectral bandwidth.

To separate protein complexes in the second dimension a gel strip from the CN-PAGE was incubated for 30 min in 25 mM Tris/HCl, pH 7.5 containing 2% SDS (w/v) and placed on top of the denaturing 12–20% linear gradient polyacrylamide gel containing 7 M urea [50]. Protein spots were stained by Coomassie Blue.

## Results

### The non-photochemical quenching in R. salina exhibits fast induction and reversibility

**Figure 1:**
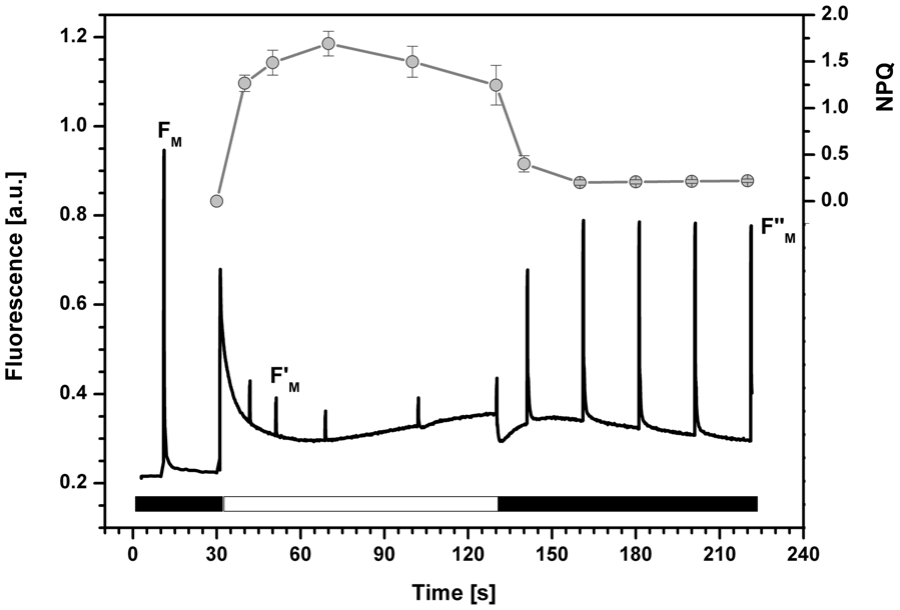
Chlorophyll a fluorescence quenching in *R. salina*. Cells were dark adapted for 20 minutes before and after irradiation. NPQ was induced by 100 s of orange actinic light (622 nm, 600 µmol m^−2^ s^−1^; white bar). Fluorescence induction curve (black line) represents a typical curve. The extent of NPQ (grey symbols, top part of the figure) was calculated as quenching of maximal fluorescence (F_M_′-F_M_)/F_M_′ for every saturating flash (n = 3); the maximal fluorescence measured after light period (F_M_″) reflects a fast recovery part of the F_M_ quenching. The value of maximal PSII efficiency calculated in dark (F_V_/F_M_) and on light (Genty parameter - φ_PSII_) was 0.79 and 0.1 respectively.

In order to elucidate the basic characteristics of NPQ in *R. salina* we measured chlorophyll a fluorescence quenching analysis by using orange actinic light preferentially absorbed by the chlorophyll *a/c* antennae (Figure 1). As it is evident from the decrease in F_M_′ (Figure 1) irradiation induced rapid quenching of maximal fluorescence during the first minute of irradiation. The calculated photochemical efficiency in the light φ_PSII_ was around 0.1 (see legend in Figure 1) suggesting the majority of quenching was caused by stimulation of non-photochemical processes and only a minor fraction of the actinic irradiance was used in PSII photochemistry. The recovery kinetics of F_M_′ (Figure 1) show NPQ is rapidly reversed in the dark resembling the typical recovery kinetics of energetic quenching (qE) as described for other organisms [4].

**Figure 2:**
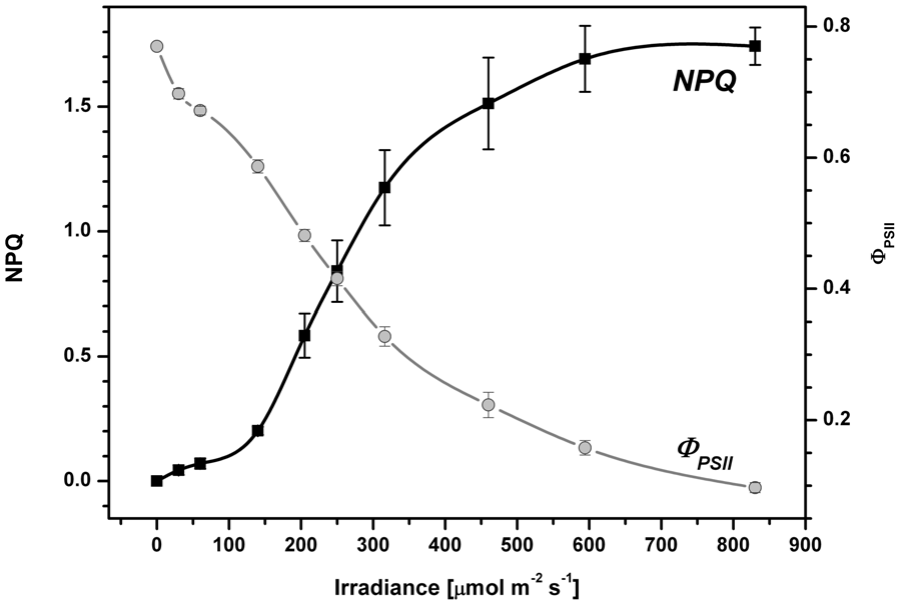
Light dependence of NPQ and the efficiency of PSII (Genty parameter) in *R. salina*. Fresh sample was used for each measurement and values were recorded always after 40 s of irradiation by orange light (622 nm). Data represent average and standard deviation for n = 3.

The non-photochemical quenching of the qE type was activated only by irradiancies above ∼150 µmol m^−2^ s^−1^ (Figure 2), lower irradiances were efficiently utilized in photosynthesis as indicated by the high efficiency of PSII photochemistry (φ_PSII_ between 0.75 to 0.55; Figure 2). The (φ_PSII_ up to ∼150 µmol m^−2^ s^−1^ was very close to the maximal PSII photochemistry observed in the dark (F_V_/F_M_ typically ∼0.79, see legend to Figure 1). The utilization of light in photosynthesis was also determined from ^14^C incorporation at different irradiancies and fitted to obtain photosynthetic parameters (see Figure S1). The maximal efficiency of photosynthesis (α) and maximal photosynthetic capacity (P_g max_) in *R. salina* cells were estimated to be 0.032±0.003 [mg C mg Chl^−1^ h^−1^ µmol^−1^ m^2^ s^1^] and 2.5±0.22 [mg C mg Chl^−1^ h^−1^] respectively. Photosynthetic rate was saturated around 150 µmol m^−2^ s^−1^ (see Figure S1). Comparison of intensity of light that stimulated NPQ (150 µmol m^−2^ s^−1^ and higher) with intensity of light that induced maximal photosynthetic rate (see Figure S1) indicates that stimulation of NPQ occurs as a feedback reaction after saturation of the Calvin-Benson cycle. In summary the mechanism of NPQ in *R. salina* is fast and is activated at light intensities exceeding the maximal photosynthetic rate.

### Non-photochemical quenching in *R. salina is* a pH dependent process

**Figure 3:**
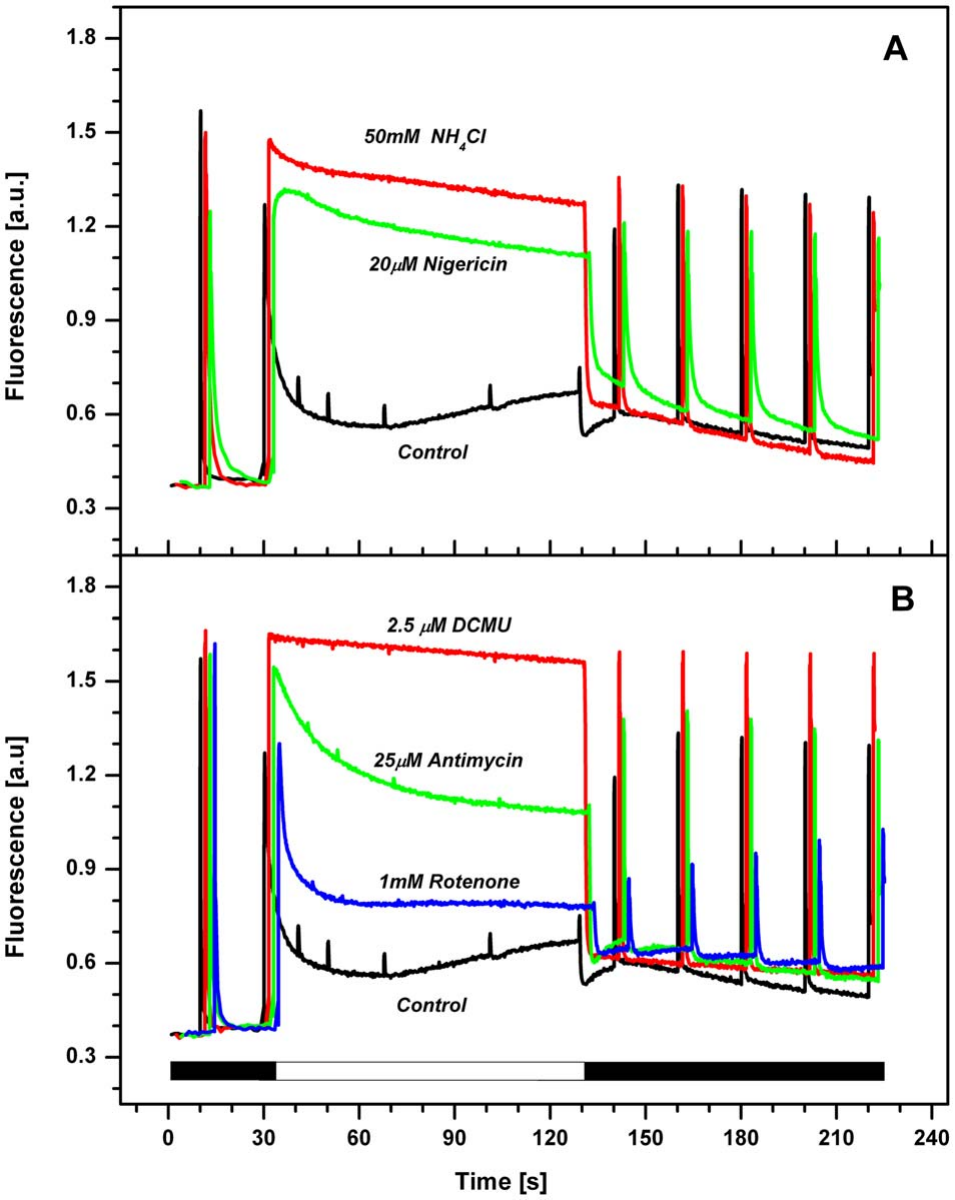
Effect of various inhibitors on NPQ in *R. salina*. Cells were dark adapted for 20 minutes and then the NPQ was induced by 100 s exposure to orange light (622 nm, 600 µmol m^−2^ s^−1^; see white bar). **A**) Effect of ΔpH uncouplers nigericin and NH_4_Cl. The maximal efficiency of PSII photochemistry (F_V_/F_M_) in the presence of uncouplers was 0.75 for control, 0.62 for nigericin and 0.68 for NH_4_Cl. **B**) Effect of inhibitors of linear and cyclic electron transport DCMU, antimycin and rotenone. All data represent typical curves aligned to the same F_o_ level.

In plants the NPQ is triggered by low lumenal pH [4], therefore we hypothesised that activation of NPQ in *R. salina* proceeds by a similar mechanism. Accordingly we found the observed stimulation of NPQ is inhibited by uncouplers that collapse the trans-thylakoid ΔpH (Figure 3A). NH_4_Cl proved to minimally affect the maximal photochemistry of PSII and inhibit NPQ quenching with greater potency than nigericin (Figure 3A). The effect of NH_4_Cl was even more pronounced in combination with nonactin; catalyzing the Δψ driven transthylakoid transport of monovalent potassium or sodium ions from lumen to stroma. We found 5 mM NH_4_Cl in combination with 2.5 µM nonactin inhibits NPQ better than 50 mM NH_4_Cl alone (data not shown). In all cases the addition of uncouplers caused total inhibition of reversible part of NPQ (F_M_′) observed during actinic light (Figure 3A). These data demonstrate a clear correlation between the activation of NPQ and lumen acidification in *R. salina*.

To explore how functional components of photosynthetic apparatus contribute to lumen acidification we used inhibitors to block electron flow at specific sites of the electron transport chain. Inhibition of PSII by DCMU (3-(3,4-dichlorophenyl)-1,1-dimethylurea) abolished the F_M_′ quenching demonstrating the dependency of NPQ on lumen acidification is related to PSII activity (Figure 3B). On the other hand, two inhibitors of cyclic electron transport around PSI, antimycin A which inhibits ferredoxin dependent cyclic electron flow and rotenone which inhibits NAD(P)H dependent cyclic electron transport, only partly affected NPQ of maximal fluorescence (Figure 3B). Nonetheless NAD(P)H dependent cyclic electron transport appears to be important for the fast recovery of fluorescence in the dark, apparent from the slower reversibility of maximal fluorescence in the dark in the presence of rotenone (Figure 3B).

### Xanthophyll cycle is not involved in NPQ in *R. salina*

We have explored possible changes in pigment composition following the long-term light stress to ascertain if *R. salina* is able to transform xanthophylls under excessive irradiation. Our data show no detectable changes in carotenoid composition after irradiation (Table 1). We have found higher relative content of alloxanthin and chlorophyll *c* in CAC antennae in comparison to intact cells (Table 1) that show the preferential incorporation of alloxanthin and chlorophyll *c* into CAC antennae. We could not detect any carotenoids involved in light-induced xanthophyll cycle (e.g., zeaxanthin, diatoxanthin) in *R. salina* cells (Table 1). This is in strict contrast to higher plants and diatoms where the violaxanthin or diadinoxanthin cycle is activated by high light [29], [51]. In *R. salina* we did not detect even trace amounts of zeaxanthin. Based on these data we conclude that the xanthophyll cycle is not involved in NPQ in *R. salina*, in line with the recently published results obtained with other cryptophyte alga, *Guillardia theta* [45].

**Table 1:** Relative pigment content in intact cells of R. salina and in their chlorophyll a/c_2_ antennae (CAC antennae).

	Chl c_2_/Chl a	Allox./Chl a	Monad./Chl a	Croco./Chl a	α-Car/Chla
**Cells - Dark**	0.55±0.03	0.74±0.06	0.16±0.02	0.15±0.01	0.18±0.02
**Cells - Light**	0.54±0.06	0.79±0.09	0.18±0.02	0.14±0.02	0.19±0.01
**CAC antennae**	1.24±0.06	1.23±0.09	0.21±0.01	0.17±0.01	0.03±0.01

Intact cells were adapted either to dark (“Cells – dark”) or exposed to intense irradiation (1500 µmol m^−2^ s^−1^) for 1 hour (“Cells – light”). CAC antennae were isolated by sucrose gradient and fraction 1 was used for analysis (see Figure 5A). Carotenoids were extracted to methanol and separated by HPLC. Note, that data represent a peak area of particular pigments relative to chlorophyll a. The total carotenoid and Chl a level was not changed significantly after high light stress.

### The NPQ in *R. salina* is connected with chlorophyll a/c antennae

**Figure 4:**
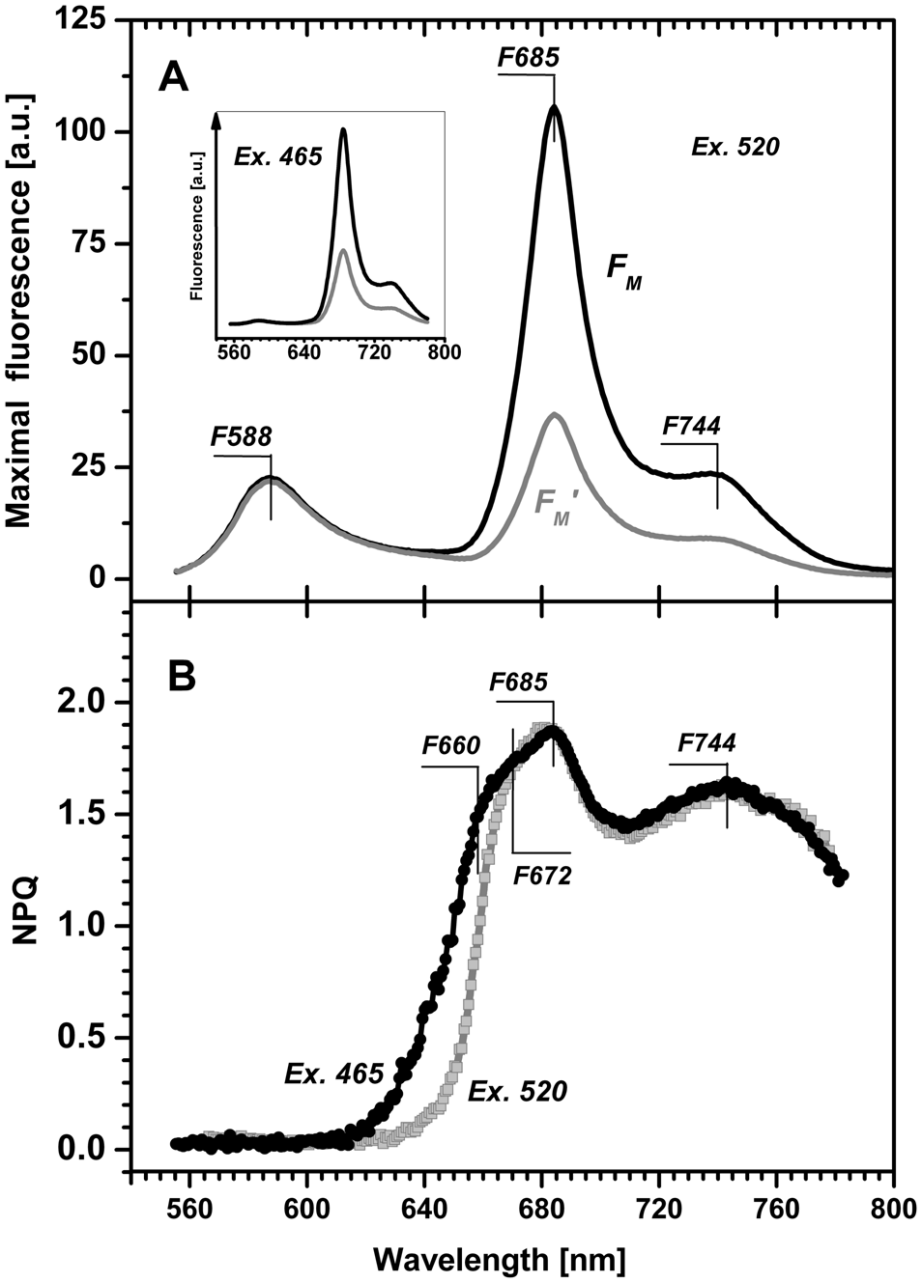
Fluorescence emission spectra (Panel A) and spectral dependence of NPQ (Panel B) of *R. salina* cells. All curves were measured using green light (520 nm, 500 µmol m^−2^ s^−1^) absorbed by phycoerythrin or blue light (465 nm, 1100 µmol m^−2^ s^−1^) absorbed by chlorophyll a. **A**) The black F_M_ spectrum induced by saturating flash (200 ms, 1100 µmol m^−2^ s^−1^) shows the fluorescence emission spectra of dark adapted cells for green light excitation at 520 nm; the grey F_M_′ spectrum induced by saturating flash (200 ms, 1100 µmol m^−2^ s^−1^) was recorded after 120 s of continuous irradiation by green light. Similar measurements using blue light excitation at 465 nm are presented in the insert. **B**) Fluorescence emission spectra described in A were used for calculation of spectral dependence of NPQ(λ) based on Stern-Volmer formalism, green light excitation at 520 nm – grey line, blue light excitation at 465 nm – black line. Data represent typical curves, characteristic maxima are marked.

As discussed earlier the fluorescence of chlorophyll *a* in *R. salina* is efficiently quenched by NPQ. However, aside from of chlorophyll *a/c* antennae cryptophytes also have phycobiliproteins firmly embedded in the lumenal space [34]. NPQ operating in phycobilisomes on the stromla side of thylakoid was recently reported in cyanobacteria (for review see [52]). To explore the possibility that phycoerythrin emission is quenched also in *R. salina* we employed the recent method of spectrally resolved fluorescence induction [47] to measure spectrally resolved NPQ in a similar way as described recently for higher plants [53]. This enabled us to measure the quenching of maximal fluorescence (F_M_′) simultaneously for several chromophores. At room temperature, two main maxima of fluorescence emission spectra can be observed in *R. salina*: the emission bands of phycoerythrin at 588 nm and chlorophyll at 685 nm (Figure 4A). We compared spectra of maximal florescence of the same sample; first when adapted to dark (F_M_ spectrum) and second after 2 min exposure to high light (F_M_′ spectrum), which allowed us to calculate spectrally dependent NPQ(λ) (Figure 4B). This result demonstrated pronounced quenching of chlorophyll *a* emissions above 640 nm but the absence of quenching of phycoerythrin emissions at 588 nm. Therefore only chlorophyll emission bands are effectively quenched between 640 nm–770 nm (Figure 4B).

Using the spectrally resolved NPQ(λ) we identified two main spectral regions of chlorophyll *a* fluorescence quenching (Figure 4B). When compared with the maximum of the PSII emission at 685 nm these regions can be assigned as quenching of the “blue” chlorophylls, with maxima between 660–685 nm, and quenching of “red-shifted” chlorophylls, with broad peak with a maxima at 744 nm. The numerical value of NPQ(λ) for the “blue” chlorophyll was ∼1.5, identical to the single wavelength calculation of NPQ measured at 690 nm (Figure 2). The maxima of NPQ(λ) found at shorter wavelengths, 660 nm, 672 nm and 685 nm (Figure 4B), correspond to the fluorescence emission maxims of chlorophyll a/c antennae and PSII of cryptophytes, as discussed later.

### NPQ in chlorophyll a/c antennae can be controlled by protonation *in vitro*

**Figure 5:**
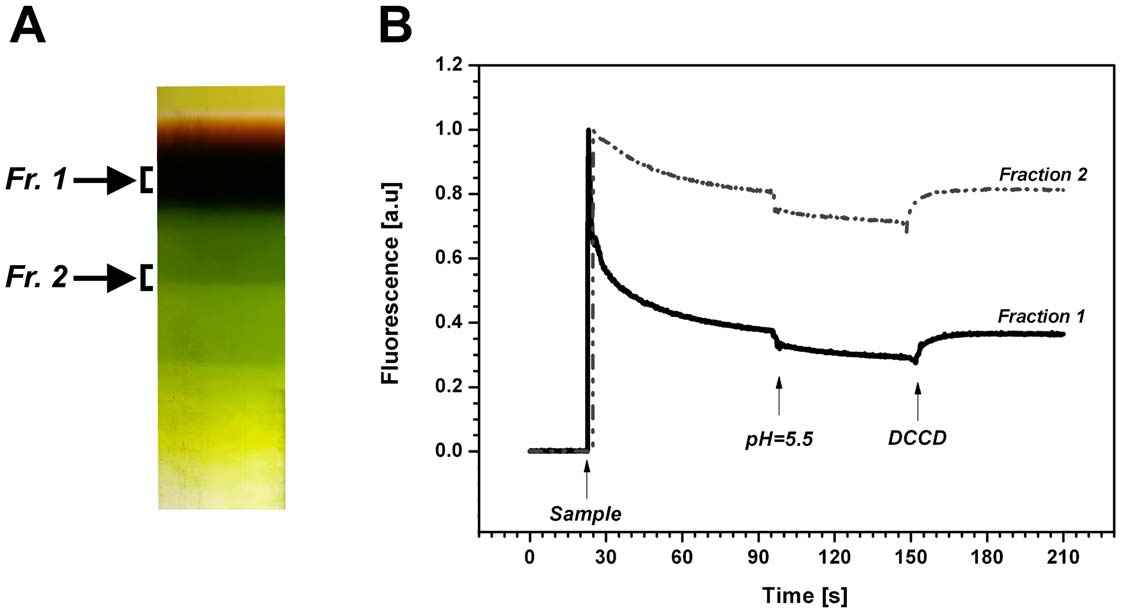
Fluorescence quenching in isolated CAC antennae. **A**) CAC proteins in a native state were isolated by ultracentrifugation in a sucrose gradient as a ‘free’ antennae (Fr. 1) or in a supercomplex with PSII (Fr. 2). See also Figures S2 and S4 for the complete figure of gradient and the spectroscopic analysis. **B**) Protein samples were diluted 10-fold to decrease concentration of dodecyl-β-maltoside – sample addition to buffer (20 mM HEPES, pH 8.0) is visible as chlorophyll fluorescence appearance (arrow ‘Sample’) that slowly decrease. After 70 s of incubation the pH in the sample was lowered from 8.0 to 5.5 (see arrow pH = 5.5) causing a fluorescence quenching. Reversibility of quenching has been confirmed by addition of 200 µM DCCD.

**Figure 6:**
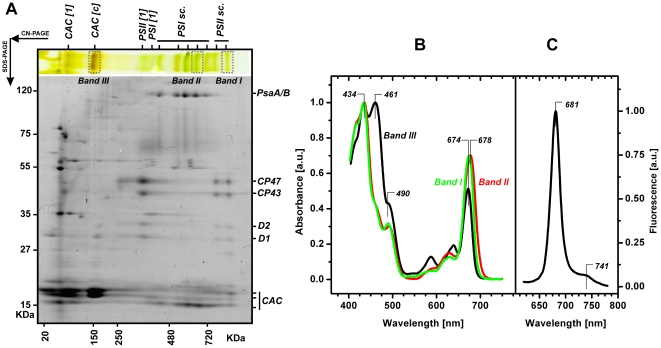
The 2D eletrophoresis of membrane protein complexes of *R. salina* and spectral characteristic of isolated bands. **A**) Membrane proteins were solubilised by dodecyl-β-maltoside and separated in a first dimension by clear-native electrophoresis (CN-PAGE). The protein complexes resolved on the CN-PAGE were further separated in the second dimension by denaturing gel (SDS-PAGE) and stained by Coomassie Blue. Position of protein complexes separated by CN-PAGE are marked as follows: CAC[1] – CAC monomers; CAC[c] – CAC oligomer; PSI[1] and PSII[1] - PSI and PSII monomers; PSI sc. and PSII sc. – supercomplexes of PSI and PSII. Proteins further resolved by SDS-PAGE are marked: CP47, CP43, D1, D2 - PSII core subunits; PsbA/B - PSI core subunits; CAC –chlorophyll a/c antenna. **B**) Absorbance spectra of Band I, II and III separated by CN-PAGE; positions of all three bands at the CN-PAGE are marked. **C**) Fluorescence emission spectra of the Band III (CAC oligomer) after excitation at 435 nm; positions of particular maxima are highlighted.

To confirm NPQ is present in chlorophyll *a/c* antenna (CAC) isolated from *R. salina*, we used sucrose density gradient ultracentrifugation to purify native complexes from solubilised membranes (Figure 5A; Figure S2). The upper dark-brown band (Fraction 1) and the higher mass green band (Fraction 2) of the sucrose gradient were analyzed by clear-native electrophoresis (CN-PAGE). In fraction 1 we resolved only one chlorophyll-protein complex (see Figure S3) and, as described later, this band was identified using 2D electrophoresis as ‘free’ CAC antennae oligomer not associated with photosystems (CAC[c] complex, Figure 6). Using the same approach, two green bands with higher mass (Fraction 2) were identified as CAC proteins in supercomplexes with photosystems (data not shown). Association of CAC antennae with photosystems in fractions 2 was further verified using absorption spectra with visible chlorophyll *c* absorption at 461 nm (Figure S4). The ability of isolated CAC proteins in both fractions to quench light energy was analysed *in vitro* using chlorophyll fluorescence (Figure 5B). First the sample was 10× diluted in a detergent free Hepes buffer (pH 8.0) to decrease the concentration of dodecyl β-maltoside to 8 µM. After sample dilution chlorophyll *a* fluorescence was significantly decreased; it should be noted this is always seen in the chlorophyll *a/b* antennae of higher plants [20]. The sample pH was then lowered to 5.5 causing further decrease in chlorophyll fluorescence (Figure 5B). Importantly, the second decrease was found to be reversible upon addition of N,N′-dicyclohexyl-carbodiimide (DCCD); a compound that specifically blocks the binding of protons to residues at light-harvesting antennae inhibiting qE in the thylakoid membrane [21]. Similar quenching was also observed in supercomplexs of CAC antennae with photosystems (Fraction 2), although there was a slower decrease in chlorophyll fluorescence after decrease in dodecyl β-maltoside concentration (Figure 5A). These results confirm NPQ occurs in the CAC antennae oligomers (‘free’ or associated with photosystems) in a process that can be controlled by their protonation.

### Oligomeric chlorophyll a/c antennae in *R. salina* form high-mass supercomplexes with PSII

To identify the chlorophyll-protein(s) responsible for NPQ in *R. salina* we used clear-native electrophoresis (CN-PAGE) to separate solubilised membranes and obtain individual protein complexes (Figure 6A). The composition of the resulting protein complexes was resolved on a second dimension; denaturing SDS-PAGE. Based on protein pattern, mobility, relative molecular masses (Figure 6A) and chlorophyll fluorescence (not shown), we identified monomeric PSI and PSII without external antennae (marked as PSI [1] and PSII [1] in Figure 6). We also found several high-mass supercomplexes of both PSI and PSII associated with antennae (PSIsc. and PSIIsc.). The most prominent pigment-protein complex separated by CN-PAGE was a chlorophyll *a/c* antenna oligomer with mass ∼150 kDa (CAC[c]; Figure 6) that appears to be composed from two different antenna proteins. The migration pattern on 2D electrophoresis has indicated this antenna complex could associate with PSII and that this association is preserved in two PSII supercomplexes detectable by CN-PAGE. Moreover, we identified a second antenna protein, CAC[1] that has a lower mass than both detectable proteins in the CAC[c] (Figure 6A). However, it should be noted that the total level of the CAC1 antenna is much lower in comparison to the number of proteins in the CAC[c] complex; thus the contribution of this antenna to the total chlorophyll quenching is negligible (Figure 6A).

All pigment-containing complexes were excised from the CN-gel and characterized by spectroscopic methods; the results obtained for PSII supercomplex (Band I, Figure 6), PSI supercomplex (Band II) and CAC[c] complex (Band III) are described in detail. The typical chlorophyll *a* absorption at 434 nm is visible in all three bands to a similar extent (Figure 6B). The characteristic peak of chlorophyll *c* absorption at 461 nm is most evident in the CAC[c] complex (Band III), but also slightly visible in PSII (Band I, Figure 6), PSI (Band II) supercomplexes demonstrating that both PSI and PSII are associated with CAC antennae; consistently with the identification of CAC in PSI/PSII supercomplexes by CN/SDS-PAGE. It should be noted that the PSI complexes had a very low quantum yield of fluorescence (data not shown) suggesting that characterized bands were not contaminated with ‘free’ chlorophyll. Additionally we observed no significant phycoerythrin absorption (∼545 nm; Figure 6B).

In comparison to PSI and PSII supercomplexes, the CAC[c] oligomer (Band III) exhibited a relatively high absorbance for chlorophyll *c* together with a high ratio of absorbance maxima at 461 nm and 434 nm. This indicates relatively high levels of chlorophyll *c* in the CAC antennae of *R. salina*. This was also confirmed by HPLC analysis of intact CAC antennae (see Table 1) that were enriched in chlorophyll c and alloxanthin relative to intact cells. As expected, the chlorophyll *a* in CAC antennae (band III) and in the PSII supercomplex (band I) have identical red absorption maxima (674 nm; Figure 6B); in contrast the PSI super-complex absorption maxima (band II) was red-shifted to 678 nm (Figure 6B).

In order to confirm that the CAC antennae of PSII represent the main locus of NPQ *in vivo* we performed room temperature fluorescence emission spectra on the CAC[c] complex (Band III; Figure 6C). This spectrum was compared to the *in vivo* spectrum of NPQ measured using whole cells (Figure 4). The CAC[c] emission has a maximum at 681 nm and a vibrational satellite at 741 nm. We therefore conclude the chlorophyll fluorescence that is quenched *in vivo* between 660–685 nm (Figure 5) originates from chlorophyll molecules located in the CAC antennae of PSII (Figure 6).

## Discussion

Cryptophyte algae represent a unique evolutionary link between red algae, which lack chlorophyll *c* but contain phycobilisomes, and diatoms, which contain chlorophyll *a/c* antennae but lack phycobiliproteins. Using *R. salina* as a model organism, we have demonstrated efficient NPQ (Figure 1) operates in cryptophytes and that the regulation of this NPQ is distinct from both red algae and diatoms. First, in strict contrast to the crucial role of the xanthophyll cycle in diatoms [17], cryptophytes have no photoprotective de-epoxidation/epoxidation cycling of xanthophyll pigments (Table 1) in line with previous results [45]. Second, we have identified specific chlorophyll *a/c* antennae of PSII as the site of NPQ in *R. salina* (Figures 4,5 and 6). Since in red algae chlorophyll *a/c* antennae of photosystem II are missing [36] and a dominant NPQ occurs rather in the reaction centres [42], [43], the cryptophytes operate new and evolutionary distinct type of NPQ.

Fast kinetics of NPQ in cryptophytes implies that it represents so-called energetic type of quenching (qE; Figure 1). This type of NPQ is already well described for higher plants [54] and is characterised by its rapid stimulation on exposure to actinic light (in tens of seconds) and fast relaxation in dark. The immediate response to changes in irradiation is due to the ΔpH dependency of qE, as the ΔpH across the thylakoid membrane is rapidly formed in the light and quickly dissipated in dark [8]. The NPQ dependency on lumen acidification has been also demonstrated in diatoms. However, there the low lumenal pH is crucial rather for triggering of the fast diadinoxanthin to diatoxanthin de-epoxidation [51] and lumen acidification alone is not sufficient to induce NPQ [17]. As *R. salina* has no light-induced xanthophyll cycle (Table 1) lumen acidification must play a critical role in NPQ induction (see Figure 3A). Therefore NPQ in cryptophytes is closely related to the qE observed in higher plants, rather than the slowly reversible qI observed in diatoms [26]. Additionally, periods of prolonged excessive irradiation therefore causes photoinhibitory damage of PSII in cryptophytes (data not shown) and does not involve an increase in the diatoxanthin pool as seen in diatoms [51].

Lumen acidification in cryptophytes appears to play a direct role in switching antennae to a quenched state by reversibly protonating CAC proteins (Figure 5). The importance of protonation for induction of NPQ has been demonstrated several times in higher plants using isolated light-harvesting antennae [20], [21], [55]. This is due to DCCD binding to carboxy amino residues located in the hydrophobic domains of light harvesting antenna that can reverse acid-induced fluorescence quenching [21]. We have performed a similar experimental procedure [55] with isolated CAC antennae, to demonstrate that the quenching of their variable chlorophyll fluorescence is pH dependent (Figure 5B). Moreover, we found the effect of low pH is reversible by using DCCD to deprotonate residues on the CAC proteins (Figure 5B), as described for light-harvesting antennae from higher plants [55]. However, the reversible part of fluorescence quenching from Fraction I (CAC proteins) and Fraction II (CAC complexes with photosystems) is small in comparison to results obtained for LHC proteins of higher plants [21], [56]. There are several possible explanations: the limited number of protonable residues in CAC, the necessity of some other factors than low pH for maximal quenching (e.g. Ca^2+^ binding to antennae [57]) or higher importance of aggregation of CAC proteins in quenching (note the relatively pronounced decrease in fluorescence before lowering pH). On the other hand, our approach confirmed an inhibitory effect of DCCD on NPQ *in vitro* (Figure 5) and also *in vivo* (Table 2), which suggests presence of pH sensing mechanisms in cryptophytes CAC antennae similarly to higher plants LHCs [21]. These results are in contrast with the situation in diatoms, where the DCCD treatment stimulates NPQ, that has resulted in speculation that the FCP proteins of diatoms may not have protonable residues [17]. The CAC proteins thus appear to be the first example of chromalveolate antennae where the protonable residues play a role in NPQ stimulation.

**Table 2:** Effect of different DCCD (N,N′-dicyclohexyl-carbodiimide) concentrations on the maximal efficiency of PSII photochemistry (F_V_/F_M_) and on NPQ.

	0 µM	2 µM	5 µM	10 µM
**NPQ**	1.17	0.64	0.74	0.37
**F_V_/F_M_**	0.75	0.71	0.73	0.65

F_V_/F_M_ values were calculated for dark adapted sample, NPQ was detected after 120 s irradiation by orange light (620 nm, 600 µmol m^−2^ s^−1^).

Our results suggest that lumen acidification is sufficient for the formation of relatively high NPQ (around 1.5) in cryptophytes, disposing of the necessity for xanthophyll de-epoxidation (Table 1). This is in contrast to green algae, in which NPQ was found to be rather weak (below 1) in a case of low violaxanthin de-epoxidation to zeaxanthin [58]. Therefore, it has been concluded that zeaxanthin is necessary for stimulation of higher NPQ values in green algae [59], [60]. In higher plants, the occasional absence of zeaxanthin can be overcome by PsbS protein that can stimulate NPQ to relatively high values (to about 1.5) even in Arabidopsis mutant without zeaxanthin [61]. PsbS protein is also required for the rapid stimulation of higher plants NPQ; its absence results in slower and less flexible NPQ, where it takes over an hour for NPQ to reach its maximal value [10]. Presently it is not known if, like higher plants, *R. salina* has a protonable PsbS-like protein. However, the rapid NPQ found in cryptophytes can result from either fast protonation of CAC antenna (see Figure 5) or higher lumen acidification. It is important to note that NPQ in *R. salina* is activated (see Figure 2) only after saturation of the Calvin-Benson cycle (above ∼150 µmol m^−2^ s^−1^, see Figure S1), which causes limited ADP regeneration [62]. In contrast the PsbS protein stimulates NPQ at all light intensities, even when Calvin-Benson cycle is not saturated. Therefore the action of only one mechanism, such as lumen acidification resulting from Calvin-Benson cycle saturation, would explain the observed light dependency of NPQ in cryptophytes; therefore rendering the presence of PsbS unnecessary.

Using spectroscopic and biochemical approaches, we localized the NPQ in *R. salina* to the CAC[c] oligomer, likely *in vivo* associated with PSII. First, we used spectrally resolved fluorescence induction [47] to calculate the *in vivo* spectral dependency of NPQ(λ) (Figure 4). These results exclude quenching of phycoerythrin fluorescence, as judging by the light-induced changes in chlorophyll emission spectra above 640 nm (Figure 4), all dissipation occurs in the chlorophyll antenna. Accordingly light absorbed by lumenal phycoerythrins is efficiently transferred to CACs, which is in line with observation that *R. salina* phycoerythrins form a very efficient light-harvesting system [63], [64]. Second, we isolated CAC[c] complexes to demonstrate that the two features of *in vivo* NPQ, pH dependency and fast reversibility, are detectable *in vitro* (Figure 5). In combination, our results lead to conclusion that NPQ in *R. salina* mainly operates in the CAC antennae of cryptophytes.

As the chlorophyll a/c antenna oligomer CAC[c] with molecular mass ∼150 kDa, has been suggested as a main NPQ locus (see previous paragraph), we analysed the organization of CAC antennae using 2D clear-native/SDS-electrophoresis (Figure 6A). This antennae complex that dissociated during native eletrophoresis from photosystems (mostly from PSII super-complexes), is composed of at least two different CAC proteins (Figure 6A) consistent with previous observations [65], [66]. Furthermore, our data show the absorption pattern of the CAC complex mirrors previous results from *Choomomas sp.* [67] and *Cryptomonas maculata* [68]. Additionally, we anticipate the CAC[c]-PSII super-complex could be homologous to *Rhodomonas CS24*, a cryptophyte alga, PSII super-complexes [69]. Using single particle analysis, these authors have shown *Rhodomonas CS24* PSII super-complexes are composed of four monomeric CAC proteins bound to one side of the PSII core dimer [69].

The oligomeric organization of antennae complexes in cryptophytes could affect properties of NPQ. For instance, in higher plants antennae trimers and minor light-harvesting antennae have been suggested to act as two different NPQ loci [53], [70]. A two-site quenching mechanism has been also suggested for diatoms [19], where a trimer of fucoxanthin-chlorophyll proteins represents typical antennae protein organization [71]. However, observations on the organization of CAC proteins in PSII supercomplexes of cryptophytes [69] suggest that CAC trimers are absent in cryptophytes, removing the possibility of a second NPQ loci. Based on these data, we speculate NPQ in *R. salina* resembles the quenching found in the minor chlorophyll a/b antennae CP24 and CP26 of higher plants [70].

It would be very interesting to compare the NPQ that we described here with the NPQ in red algae, as the LhcR antennae of red algae are the closest relatives to CAC antennae of cryptophytes [36]. Since the NPQ in red algae showed several similarity with NPQ in cryptophytes (e.g. pH-dependency [42], [43], low importance of xanthophylls cycle [72], [73], [74]), it indicates that also the NPQ mechanisms seems to be evolutionary related. Here we have demonstrated that NPQ cryptophytes represents a novel class of effective NPQ that proceeds on a level of chlorophyll a/c antennae (CAC) and not in phycobiliproteins and its important properties differ significantly from NPQ described in diatoms and in higher plants. For example, the typical carotenoid quenchers found in higher plants (lutein and zeaxanthin) or in diatoms (diatoxanthin) are absent in cryptophytes. The observed absence of NPQ in phycobiliproteins means that periods of excessive irradiation absorbed by phycobiliproteins have to be ‘managed’ by its rapid transfer to CAC antennae for a safe dissipation. Thus, the cryptophytes, and in particular *R. salina*, represents a new model organism for the study of photoprotection and NPQ, which is going to be facilitated by the imminent completion genome sequence for a cryptophytes representative.

## Supporting Information

Figure S1 Light response curve of photosynthetic rate (P_g_) in *R. salina* measured from ^14^C incorporation rate. The typical data set (circles) is shown, line is a fit of experimental data by hyperbolic tangent. The average values calculated from the fit were: maximal efficiency of photosynthesis 0.032±0.003 [mg C mg Chl^−1^ h^−1^ µmol^−1^ m^2^ s^1^]; photosynthetic capacity 2.5±0.22 [mg C mg Chl^−1^ h^−1^].
(TIF)

Figure S2 *R. salina* thylakoids solubilised with dodecyl-β-maltoside and separated by centrifugation on a sucrose gradient. Positions of particular bands used for i*n vitro* measurements of fluorescence (Figure 5) and for the detection of absorbance (Figure S4) are marked.
(TIF)

Figure S3 Clear-native electrophoresis of fraction I isolated from sucrose gradient (Figure S2). Fraction I was separated by 4–12% gradient polyacrylamide gel as described in material and methods. Resulting gel was scanned in true colours by Canon CanoScan 8800F scanner (colour) and in high-resolution gray scale mode (DIA) using LAS 4000 (Fujifilm Life Science, USA). Finally, chlorophyll fluorescence was detected using LAS 4000 with 460 nm excitation wavelength and 670 nm long pass filter.
(TIF)

Figure S4 Absorbance spectra of protein fractions containing CAC antennae isolated on a sucrose gradient. See text for details.
(TIF)
