# Supplementary figures and images for: Non-Photochemical Quenching in Cryptophyte Alga Rhodomonas salina Is Located in Chlorophyll a/c Antennae
Source: PLoS One. 2012 Jan 3;7(1):e29700. doi: 10.1371/journal.pone.0029700 (PMC3250475; doi:10.1371/journal.pone.0029700)

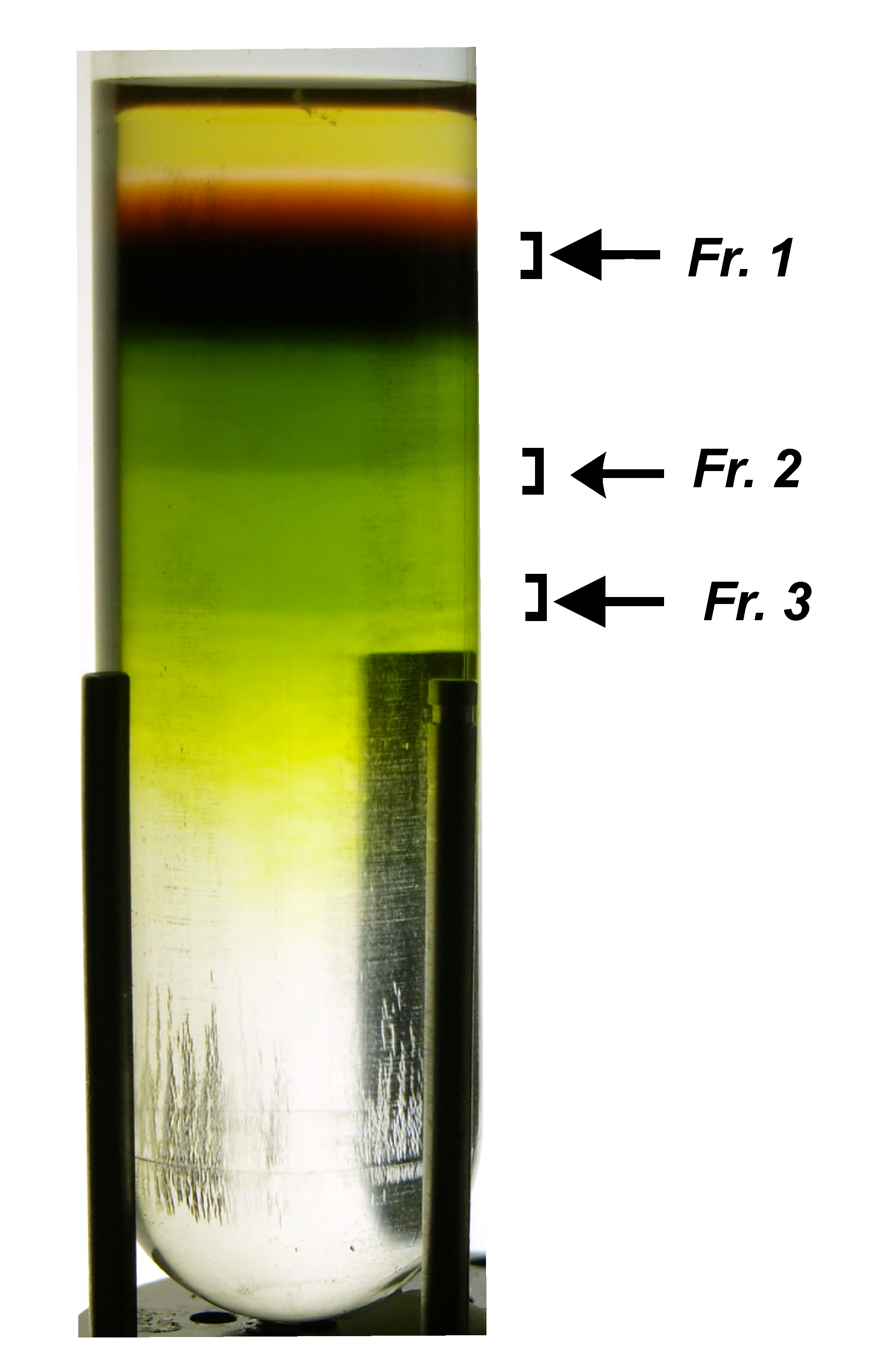

Supplement: Figure S2 — R. salina thylakoids solubilised with dodecyl-β-maltoside and separated by centrifugation on a sucrose gradient. Positions of particular bands used for in vitro measurements of fluorescence (Figure 5) and for the detection of absorbance (Figure S4) are marked. (TIF) [file pone.0029700.s002.tif]

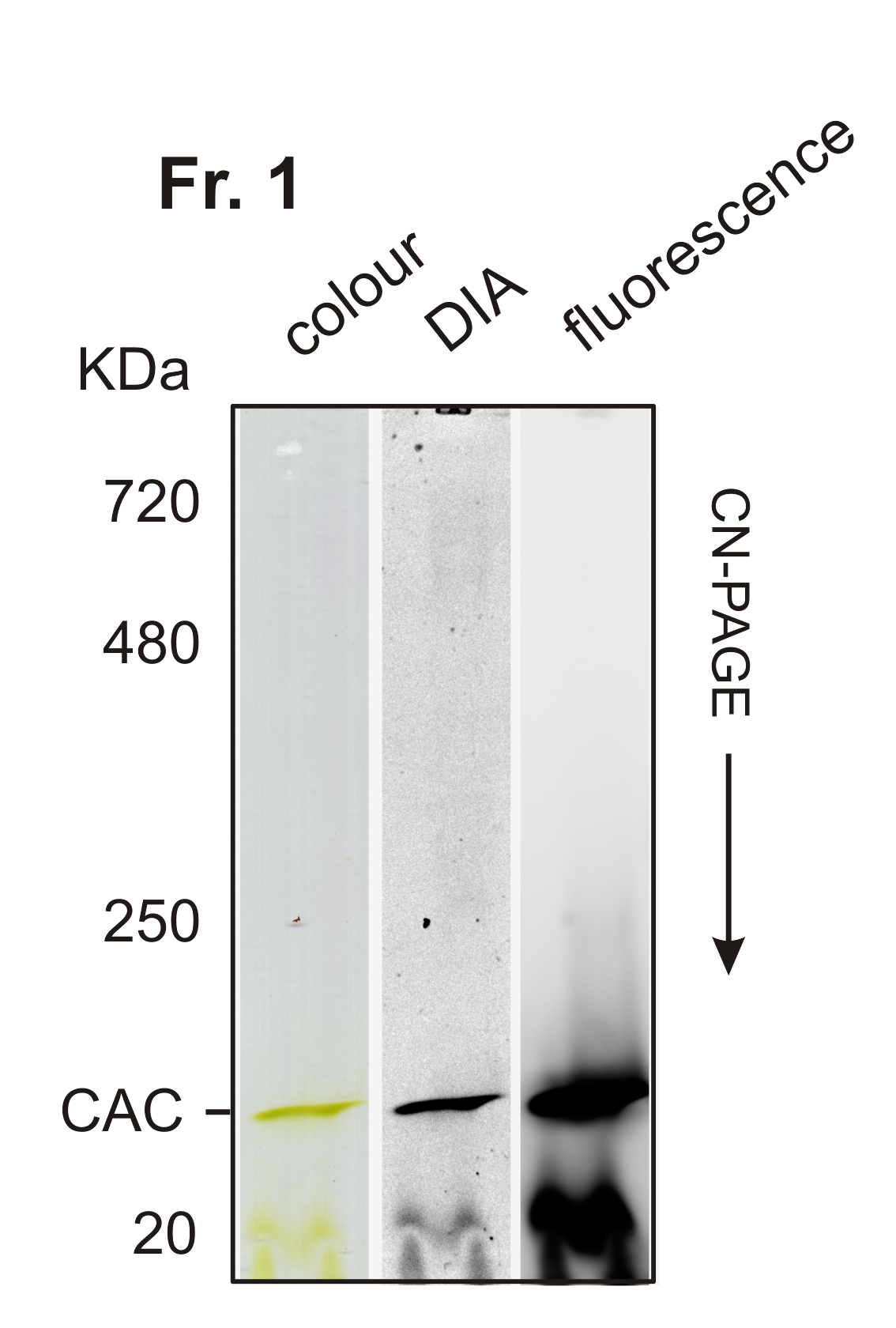

Supplement: Figure S3 — Clear-native electrophoresis of fraction I isolated from sucrose gradient (Figure S2). Fraction I was separated by 4–12% gradient polyacrylamide gel as described in material and methods. Resulting gel was scanned in true colours by Canon CanoScan 8800F scanner (colour) and in high-resolution gray scale mode (DIA) using LAS 4000 (Fujifilm Life Science, USA). Finally, chlorophyll fluorescence was detected using LAS 4000 with 460 nm excitation wavelength and 670 nm long pass filter. (TIF) [file pone.0029700.s003.tif]
